# The National Surgical, Obstetric, and Anesthesia Plan (NSOAP): Recognition and Definition of an Empirically Evolving Global Surgery Systems Science: Comment on "Global Surgery – Informing National Strategies for Scaling Up Surgery in Sub-Saharan Africa"

**DOI:** 10.15171/ijhpm.2018.87

**Published:** 2018-09-16

**Authors:** Gregory L. Peck, Joseph S. Hanna

**Affiliations:** Department of Surgery, Robert Wood Johnson Medical School, Rutgers University, New Brunswick, NJ, USA.

**Keywords:** Surgery Systems Science, Participatory Action Research, Dissemination/Implementation Science, Colombia, GSRU

## Abstract

In 2015, the Lancet Commission on Global Surgery (LCoGS) working groups developed a National Surgical, Obstetric, and Anesthesia Plan (NSOAP) framework to guide national surgical system development globally predicated on six data points (indicators) which can assess surgical systems. Zambia as well as other subSaharan Africa (SSA) countries have forged ahead in designing and implementing interventions based on LCoGS indicators collected to inform NSOAP. Concurrently, the Zambian team and others have recognized the need for rigorous scientific inquiry to assess and iteratively improve upon the NSOAP process and outputs. Based on the Zambian experience, as well as that of ours in Colombia, we have identified "core principles" through convergent works which inform a scientific framework through which NSOAP can be evaluated. We propose that when contextualized, participatory action research (PAR) and dissemination and implementation science are methodologies upon which a robust framework can be developed to achieving objective and iterative NSOAP evaluation, and ultimately universal health coverage as envisioned by the World Health Organization (WHO).

We read with great interest the editorial entitled “Global Surgery – Informing National Strategies for Scaling Up Surgery in Sub-Saharan Africa”and commend Gajewski and colleagues for their innovation and progress in SSA which has set an example for the larger global surgery scientific community.^1^ The authors seek to evaluate and frame the work being done to strengthen the surgical workforce in sub-Saharan Africa (SSA) within the context of the goals established by the Zambian National Surgical, Obstetric, and Anesthesia Plan (NSOAP). They do so within the larger context of the global surgical need which informs the scale of the problem and discuss two different, yet complimentary and in the present case co-dependent, research models that may be considered to address the burden assessment and proposed interventions. This self-critical analysis is an essential process through which the global surgery scientific community can begin to understand the efficacy and impact of assessment and strengthening activities in the short, medium, and long-term.

NSOAP development has been predicated upon an empirically defined process of “indicator” assessment which is used to inform preparedness, delivery and cost. Six indicators were described and supported with available data and systems modeling in the seminal Lancet Commission on Global Surgery (LCoGS) publication, Global Surgery 2030: evidence and solutions for achieving health, welfare, and economic development.^2^ Infrastructure, Workforce, Service Delivery, Finance/Governance, and Information Management are the health systems building blocks of NSOAP informed by the indicators. In the interim, multiple groups around the globe have invested substantial resources and energy in pursuing these indicators on several continents. Recent analysis of the validity of the LCoGS indicators suggests that they are effective in providing a meaningful and objective assessment of a surgical system’s capacity.^3^ However, the process through which NSOAP is developed is self-defined and generated based on empiric observation and in-country experience by identified stakeholders. The present authors provocatively point out that there is no “strong empirical base” for this process and opine that perhaps without this, “national surgical plans will be unrealistic or remain aspirational.”

Following a concise summary of global NSOAP progress, the authors reflect on the processes by which they have arrived at their present *research agenda* ie, methodology and development by describing the inception of COST-Africa and its iterative progeny SURG-Africa. They then introspectively analyze the barriers and opportunities which they have encountered working towards the effective development, assessment and intervention of NSOAP in Zambia. Ultimately, they strive to synthesize a rational way forward incorporating lessons learned by ‘linking knowledge-building with action as its happening.’

On deeper analysis of the experience and perspective Gajewski and colleagues have presented, and comparing it to our own and others, it is clear that, as with other groups engaged in surgical system assessment and strengthening, the present authors have worked through several aspects of global surgery research which as of yet remain undefined and in evolution. Furthermore, as with other groups, serendipity has punctuated their work to reveal core principles central to the science of global surgery. We believe that the independent convergence of distant groups upon similar revelations suggests identification of meaningful truths that inform the developing discipline of *global surgery system science*. Herein, we augment and expand on the authors experientially identified “*core principles*” (Table 1) based on our work in Latin America.^4-6^

**Table 1:** Proposed Five Global Surgery Science Core Principles Experientially Identified

**Principle 1**	The specialized “metrics language” of the LCoGS indicators is essential to facilitate effective global communication and discussion.
**Principle 2**	Mixed methodologies of research such as PAR and DIS may be better suited than traditional approaches to assess highly complex and interconnected systems with a myriad of stakeholder inputs and interactions.
**Principle 3**	As research methods are developed and or adapted from other fields to address various aspects of surgical system assessment and strengthening, rigorous scientific methodology is needed to assess and inform these evolving methods.
**Principle 4**	Highly effective trans-national teams with stakeholder representation across disciplines and professions are needed to address the global surgical burden in partnership with individual communities to establish durable and effective mechanisms of iterative NSOAP development.
**Principle 5**	The nucleus of highly distributed global surgery teams is the “learner,” who through participation evolves into a *systems aware global surgeon* desirous and capable of addressing the social responsibility of resolving the global surgical burden, moving the world closer to *universal health coverage.*

Abbreviations: LCoGS, Lancet Commission on Global Surgery; DIS, Dissemination and Implementation Science; NSOAP, National Surgical, Obstetric, and Anesthesia Plan; PAR, participatory action research.

First, the authors begin analysis of the scientific aspect of their work in SSA by establishing the context within which they wish to engender change. While it is understood by all that surgical systems are highly complex and adaptive, how best to tackle multi-level assessment and change has remained a daunting task until most recently. In proposing six core indicators to describe surgical systems, the LCoGS has demystified the process through which complexity is given order and simplicity. Furthermore, this specialized “metrics language” is essential to facilitate effective communication and discussion. The implicit “truth” of these observations is reaffirmed in the broad acceptance and incorporation of the LCoGS indicators into global surgery efforts across continents and is the first *core principle*. Insightfully, the authors look beyond this to ask the following: how do “we” study the mechanism and efficacy of the intended intervention and the effects of the anticipated change that are proposed within this context?

This multilevel intellectual inquiry requires definition of the “levels” at which innovation and change is being proposed, followed by identification and or development of appropriate methodologies to study the question being asked. In other words, the methodology must be contextualized. Therein lies the second *core principle* broached within the present editorial: traditional scientific methods may not be well suited to study health system strengthening and the effects on health and wellbeing from a surgical perspective? By stating that the absence of an empirical base from which to study and understand national surgical plans may result in ultimate failure, the authors implicitly question the validity of traditional scientific methods. We propose that when viewed through the lens of traditional quantitative scientific methods, the task may indeed appear “unrealistic or remain aspirational.”

Herein we find one of the first ‘serendipitous’ events in the Zambian experience. In the process of beginning to “define” an intervention to address the workforce shortage, the lack of objective data to describe the contributing factors engendered significant potentially crippling debate described by the authors as “politicization.” Fortuitously, in 2015 the Surgical Society of Zambia was invited to join a national working group to give voice to the “local community” of providers as an NSOAP was being drafted. Concomitantly, on the other side of the globe, our engagement in Latin America started at the “grass-roots” level working with learners and providers on the ground to identify processes by which the indicators could be collected in Colombia, specifically in Medellin and Cali. This approach was chosen out of necessity as ministry level partnership had not yet developed, and we wished to obtain a granular understanding of indicators 1, 5, and 6 which historically has been difficult to achieve given the aggregate nature of ministry level data. Intuitively, a *participatory* grass-roots approach seemed to address the community’s needs.

Taking these two experiences together and reviewing available research methodologies, it becomes clear that *participatory action research* (PAR) is well suited to address these questions in the present context. PAR is a qualitative research methodology through which the stakeholders, ie, underserved community members, become active participants in all aspects of the research process “for the primary purpose of imparting social change; a specific action (or actions) is the ultimate goal.”^7^ PAR involves the action researcher and a community seeking to improve its circumstances. *Apropos* to surgical system assessment and strengthening is the PAR philosophy as articulated by Attwood: “the concept that people have a right to determine their own development and recognizes the need for local people to participate meaningfully in the process of analyzing their own solutions, over which they have (or share, as some would argue) power and control, in order to lead to sustainable development.”^8^

Recognizing the importance of PAR in their work to address the lack of an empiric baseline, Gajewski and colleagues astutely define critical processes which should be applied to surgical system strengthening. This list is a contextualization of the components of the PAR process which have been previously described.^9^ MacDonald summarizes the seven components as follows:

1. The issue is defined, analyzed and solved by the “community.”
2. Radical change and improvement in individual lives is the ultimate goal.
3. Requires full and active participation of community members.
4. Participants to include the breadth of those affected in all capacities.
5. Creation of awareness of own resources to be mobilized.
6. Community participation allows for a more realistic understanding of the problem.
7. Inherent to the model, the “community researcher” leads discovery and directly benefits from the findings which fosters “ownership.”

Although not stated in the authors process list though clearly intimated, the last two points are critical in establishing *understanding* and *sustainability*.

While PAR appears particularly suited to the evaluation and development of NSOAP, an objective methodology is needed to assess the efficacy of its findings, recommendations, and outcome of implemented changes. Herein the third *core principle* becomes evident: as research methods are developed and or adapted from other fields to answer various aspects of surgical system assessment and strengthening, rigorous scientific methodology is needed to assess and inform these evolving methods. We propose that this “second level” of research is best accomplished through the maturing field of Dissemination and Implementation Science (DIS). *Implementation strategy* has been defined as *methods or techniques* used to enhance the adoption, implementation, and sustainability of a program or practice.^10^ Proctor et al elaborate on this point and state, “an implementation strategy must have *study design that elicits measurability and reproducibility…much of which comes by naming, defining, and operationalizing particular strategies.”*^11^ Consequently, we can know how, when, why, and where an implementation strategy is likely to be effective.

Within DIS, there are several frameworks, which can be utilized depending on the context. The Consolidated Framework for Implementation Research (CFIR) appears well suited to NSOAP science.^12^ CFIR addresses the need to evaluate not only ‘summative outcomes but also formative outcomes to assess the extent to which implementation is effective in a specific setting, prolongs sustainability, and promotes dissemination into other settings.’^13^ Our application of the CFIR to ongoing efforts in Latin America is summarized and contextualized in Table 2. Consistent with our implementation of PAR, our CFIR construct incorporates the “community” in the Global Surgery Research Unit (GSRU), which is the learner community-based research team working towards indicator collection, and ultimately NSOAP in Colombia and other parts of Latin America and Caribbean.

**Table 2:** Colombia’s Operationalization of CFIR: 5 Domains for NSOAP and LCoGS Indicator Data Collection, Analysis, and Interpretation

**Variable**	**Definition**
Characteristics of the intervention	An adaptive qualitative methodology that guides iterative quantitative WDI data collection, analytics, and interpretation by GSRUs grassroots implementers of WDI data collection and NSOAP with 30 day global surgery research unit (GSRU) intervals
Individuals	HIC/LMIC research fellows and HIC/LMIC clinical faculty (GSRUs) at the grassroots level joined through a transnational institutional MoU
Inner setting	HIC/LMIC institutions with Colombia MoH, LCoGS, WHO, World Bank; current national health care agenda and policy regarding NSOAP framework
Outer setting	Public and private hospitals requiring formal and multi-sectoral support in providing timely, safe, and affordable surgical, obstetrical, and anesthesia care with no or an iteratively developing NSOAP
Implementation process	A grassroots effort in NSOAP that hinges on WDI data collection, analytics, and interpretation, and results in measured improvement of surgical preparedness, delivery, and affordability indicators at the population level from early implementation outcomes in Colombia

Abbreviations: GSRU, global surgery research unit; NSOAP, National Surgical, Obstetric, and Anesthesia Plan; LCoGS, Lancet Commission on Global Surgery; CFIR, Consolidated Framework for Implementation Research; WDI, World Development Indicators; MoU, memorandum of understanding; MoH, Ministry of Health; HIC, high-income country; LMIC, low- and middle-income country.

Expanding upon the SSA experience, which mirrors our own, we have identified two additional core principles through convergent tracks of investigation. The fourth *core principle* empirically recognized is that addressing the global surgical burden cannot be done by “groups” of individuals. Instead, highly effective trans-national networks (or teams) with stakeholder representation across disciplines and professions are needed to initiate these processes and partner with individual communities, ie, states and nations, to establish durable and effective mechanisms of iterative NSOAP development through PAR and evaluated by DIS. Assessment of the most effective networks through team science is likely to yield important lessons vital to achieving global universal health care.^14^

Finally, recognition that the nucleus of these highly distributed teams is the “learner,” who through participation evolves into a *systems aware global surgeon* desirous and capable of addressing the social responsibility of resolving the global surgical burden, moving the world closer to *universal health coverage* is the fifth *core principle*. The recent proliferation of learner-initiated national and international organizations^15,16^ dedicated to addressing the global surgical burden reflects their energy and enthusiasm, which ideally should be harnessed and directed through dedicated mentorship. Learners are actively seeking education in global surgery issues, and training in context appropriate research methods. In our own program, students have become the primary effectors of implementation and change in partnership with the community through the GSRU construct applied in Latin America. Most gratifyingly, we have observed their increasing primary intellectual contributions to the research agenda, evidence of their evolution and development.

## Conclusion

The SSA and Latin American implementation strategies offer convergent experiences from which empiric observations can be made, and we propose, generalized to the field of global surgery research. The collective experience working towards NSOAP development through LCoGS indicator data collection has revealed the need for adoption of mixed methods research incorporating quantitative and qualitative approaches using science developed in other disciplines including PAR, DIS and team science. We believe that these methodologies will facilitate a data driven, iterative approach to NSOAP creation and implementation and will inform the future formalization of global surgery systems science and development of the learner as a systems aware global surgeon.

## Ethical issues

Not applicable.

## Competing interests

Author declares that he has no competing interests.

## Authors’ contributions

Both authors participated in the drafting, critical writing, conceptualization, and final manuscript writing.
